# POLR2A Promotes the Proliferation of Gastric Cancer Cells by Advancing the Overall Cell Cycle Progression

**DOI:** 10.3389/fgene.2021.688575

**Published:** 2021-11-25

**Authors:** Qiuyu Jiang, Jinyuan Zhang, Fang Li, Xiaoping Ma, Fei Wu, Jiyu Miao, Qian Li, Xiaofei Wang, Ruifang Sun, Yang Yang, Lingyu Zhao, Chen Huang

**Affiliations:** ^1^ Institute of Genetics and Development Biology, Translational Medcine Institute, Xi’an Jiaotong University, Xi’an, China; ^2^ Department of Oncology, The Second Affiliated Hospital, Xi’an Jiaotong University, Xi’an, China; ^3^ Department of Hematology, The Second Affiliated Hospital, Xi’an Jiaotong University, Xi’an, China; ^4^ Department of Gastroenterology, The First Affiliated Hospital of Xi’an Medical University, Xi’an, China; ^5^ Biomedical Experiment Center, Xian Jiaotong University, Xi’an, China; ^6^ Department of Toxicology and Sanitary Analysis, School of Public Health, Xi’an Jiaotong University, Xi’an, China

**Keywords:** PolR2a, gastric cancer, transcriptional regulation, cell cycle, oncogene

## Abstract

RNA polymerase II subunit A (POLR2A) is the largest subunit encoding RNA polymerase II and closely related to cancer progression. However, the biological role and underlying molecular mechanism of POLR2A in gastric cancer (GC) are still unclear. Our study demonstrated that POLR2A was highly expressed in GC tissue and promoted the proliferation of GC *in vitro* and *in vivo*. We also found that POLR2A participated in the transcriptional regulation of cyclins and cyclin-dependent kinases (CDKs) at each stage and promoted their expression, indicated POLR2A’s overall promotion of cell cycle progression. Moreover, POLR2A inhibited GC cell apoptosis and promoted GC cell migration. Our results indicate that POLR2A play an oncogene role in GC, which may be an important factor involved in the occurrence and development of GC.

## Introduction

Gastric cancer (GC) is one of the most common malignant tumors in the digestive system. In accordance with global cancer statistics in 2018, the incidence of GC ranked fifth, and the mortality rate ranked third in malignant tumors worldwide, while its incidence in East Asia ranked first in the world (Bernecky et al., 2016). In recent years, the incidence of GC has increased year by year, and the burden of social and health expenditure has also increased. Surgery combined with radiotherapy and chemotherapy is currently the main method for the treatment of GC. However, because the early symptoms of GC are hidden and most of them are in the middle and late stages of diagnosis, the 5-years survival rate is still less than 20% (Bertoli et al., 2013; Bray et al., 2018). General resistance to chemotherapy drugs is also one of the main reasons for poor efficacy. GC is a heterogeneous disease, the differences of epidemiological and histopathological between countries are the main cause of cancer-related deaths (Chapman et al., 2008). The occurrence and development of GC is a multi-step process involving many genetic and environmental factors. According to histopathological classification, it can be divided into adenocarcinoma, squamous cell carcinoma, and adenosquamous carcinoma. The currently widely used histopathological classification has gradually become difficult to adapt to the needs of clinical individualized diagnosis and treatment. With the development of gene chip and next-generation sequencing technology, the study on GC has entered the molecular level (Coffman, 2004; Chia and Tan, 2016; Clark et al., 2016). Therefore, understanding the molecular mechanism of the occurrence and development of GC is extremely crucial for its diagnosis and treatment.

RNA polymerase (RNAP) is the most critical enzyme in the transcription process, and it plays an extremely significant role in gene expression. Unlike prokaryotic cells where there is only one RNAP responsible for all mRNA, rRNA, and tRNA synthesis, eukaryotes have three different RNAPs, which specifically transcribe different genes to produce different products. Among them, RNAP Ⅱ is involved in the transcription of all protein-coding genes, snoRNA and some snRNA, and it is located in the nucleoplasm of cells (Das et al., 2004). RNAP Ⅱ requires the participation of a variety of transcription factors when initiating transcription, in order to form an active transcription initiation complex that binds to the promoter, so there are more studies on it (Errico, 2015). RNAP Ⅱ consists of 12 subunits, of which the largest subunit is encoded by the POLR2A gene (Gao and Liu, 2019). The human POLR2A gene is located on chromosome 17p13.1. The carboxyl end of its product has a 7-amino acid common repeat sequence of Tyr-Ser-Pro-Thr-Ser-Pro-Ser, called the carboxy-terminal domain (CTD), which is essential for polymerase activity and also necessary for maintaining cell activity. The CTD structure is unique to RNAP Ⅱ, not in RNAPⅠ and RNAP Ⅲ (Griesenbeck et al., 2017; Harlen and Churchman, 2017; Hou et al., 2019). In addition, this subunit combines with several other polymerase subunits to form the DNA binding domain of the polymerase, which is the groove where the DNA template is transcribed into RNA (Hsin and Manley, 2012).

Studies have shown that, the expression of POLR2A was significantly lower expressed in normal elderly and Werner syndrome patients compared with young donor cells, suggesting that POLR2A may be involved in regulating cell senescence (Jeronimo et al., 2013). Moreover, Hou et al. proved that POLR2A is the main target gene down-regulated after Xeroderma pigmentosum group A (XPA)-binding protein 2 (XAB2) is depleted (Hou et al., 2019). XAB2 depletion leads to massive loss of POLR2A and triggers a cascade of global transcription and cellular senescence (Jeronimo et al., 2016). Repeated mutations of POLR2A were found in samples lacking known meningioma driver genes, suggesting that POLR2A may play a role in tumorigenesis (Koumenis and Giaccia, 1997). Furthermore, in triple negative breast cancer (TNBC) and colorectal cancer (CRC), inhibition of POLR2A has a selective inhibitory effect on the growth of tumors with loss of POLR2A hemizygotes. Homozygous deletion of POLR2A is lethal in human cells, shows that POLR2A is essential for cell survival (Kurokawa et al., 2017; Gao and Liu, 2019). However, the biological effects and molecular mechanisms of POLR2A in GC cells are rarely studied.

In the present study, we investigated the role and the molecular mechanism of POLR2A in regulating GC cell proliferation and migration. Our study showed that POLR2A was highly expressed in GC tissues, and promoted the proliferation of GC *in vivo* and *in vitro*. Futhermore, POLR2A significantly promoted the overall cell cycle progression in GC by facilitating cyclins and CDKs transcription. POLR2A also inhibited GC cell apoptosis and accelerated GC cell migration. These results indicate that POLR2A plays the role of oncogene in GC and is expected to become a potential therapeutic target for GC.

## Materials and Methods

### Collection of Human GC Tissue Samples

GC tumor and adjacent normal tissue samples from 39 patients were randomly collected from the First Affiliated Hospital of Xi’an Jiaotong University, PR China. The tissues were divided into two parts, one part was fixed in 4% paraformaldehyde for paraffin embedding, and the other part was stored at −80°C for further analysis. The clinicopathological characteristics of the patients are summarized in Supplementary Table S1. The consent of each patient was obtained before the samples were collected. The study was approved by the Biomedical Ethics Committee of the Medical Department of Xi'an Jiaotong University.

### Cell Culture

Human GC cell lines MKN-28, MKN-45, AGS, SGC-7901, BGC-823 and normal gastric mucosal epithelial cell line GES-1 were obtained from Cell Bank (Genechem, Shanghai, China). All the cell lines had been authenticated by the Cell Bank. The cells had been tested for mycoplasma before all experiments began. All cell lines were cultured in RPMI-1640 or DMEM (Basalmedia, Shanghai, China) medium containing 10% fetal bovine serum (FBS, Biological Industries, Israel) and 1% penicillin-streptomycin solution (solarbio, Beijing, China), and incubated in a humidified incubator at 37°C with 5% CO_2_.

### Immunohistochemistry

GC tissues were fixed in 4% paraformaldehyde, then embedded in paraffin. The tissues were cut to a thickness of 5 microns with a microtome. The sections were treated with xylene to deparaffinize, and graded alcohol treated for hydration, and then antigen retrieval was applied. The rabbit SP kit (rabbit streptavidin-biotin detection system, OriGene, United States) was performed to break the endogenous peroxidase and block according to the manufacturer’s instructions. The slides were incubated with the POLR2A specific primary antibody overnight at 4°C, and then the secondary antibody was incubated at room temperature, and the horseradish enzyme-labeled streptavidin working solution was processed. DAB staining kit (OriGene, United States) and hematoxylin were used for staining. The slides were dehydrated and sealed prior to microscopic analysis (Media Cybernetics, United States). The primary antibody for IHC are listed in Supplementary Table S2.

### RNA Extraction and Quantitative Real-Time Polymerase Chain Reaction

Total RNA was extracted from cell lines or frozen tissue using TRIzol reagent (Genestar, Shanghai, China) according to the manufacturer’s instructions. RNA sample concentrations were measured using Spectrophotometer (DeNovix, United States) spectrophotometrically. Complementary DNA (cDNA) was synthesized by cDNA Synthesis Kit (Yeasen, Shanghai, China) according to the manufacturer’s instructions. qRT-PCR was performed using the SYBR Green PCR kit (Yeasen, Shanghai, China). The three-step method was applied as the amplification procedure. First, pre-denatured at 95°C for 5 min. Then denatured at 95°C for 10 s, annealed at 55–60°C for 20 s, and extended at 72°C for 20 s, this step requires 40 cycles. The default setting of the instrument was adopted for the melting curve stage. The primers are listed in Table S3. All qRT-PCR reactions for each sample were performed in triplicate, using the IQ5 multicolor qRT-PCR detection system (Bio-Rad, United States). GAPDH were used as control for messenger RNA (mRNA). The 2^−ΔΔCt^ method was utilized for the qRT-PCR analysis.

### Protein Extraction and Western Blotting

Human GC cells were lysed with precooled RIPA buffer (Pioneer, Xi’an, China) with protease inhibitor (Pioneer, Xi’an, China) for 30 min on ice. The samples were then centrifuged (14,000 rpm for 20 min at 4°C), supernatants were collected. The protein concentrations were determined by the BCA quantification kit (Fdbio, Hangzhou, China). Protein samples (20 μg) were separated by sodium dodecyl sulfate-polyacrylamide gel electrophoresis and transferred to polyvinylidene difluoride membranes (Merck Millipore, Germany). The membranes were blocked with 5% skim milk for 1 h at room temperature and incubated with specific primary antibodies at 4°C overnight. Before and after incubation with the secondary antibody for 1 h at room temperature, the membranes were thoroughly washed with TBST buffer containing Tween-20 (Fdbio, Hangzhou, China) for 10 min, a total of three times. Finally, the membranes were exposured with an enhanced chemiluminescence detection kit (Fdbio, Hangzhou, China) to show protein bands. GAPDH was performed as an internal control. Imaging signals were acquired and analyzed by ChemiDoc™ Touch (Bio-Rad, United States). The primary and secondary antibodies used are listed in Supplementary Table S2.

### siRNA Synthesis, Plasmid Construction and Transfection

Small interfering RNAs (siRNAs) targeting human POLR2A were designed and generated by GenePharma (Shanghai, China). A sequence with no homology to mammalian genes was used as a negative control (NC, GenePharma). The sequences are listed in Supplementary Table S4. The full-length human POLR2A cDNA was cloned into the pCMV2-GV219 vector (Genechem, Shanghai, China) to construct its overexpression plasmid. After culturing MKN-45 and SGC-7901 cells in the plate for 24 h, siRNA or plasmids were transfected into the cells using jetPRIME® *in vitro* DNA and siRNA transfection reagent (PolyPlus, France) according to the manufacturer’s protocol and the usage of transfection reagent is listed in Supplementary Table S4.

### MTT Assay

MKN-45, SGC-7901 cells were seeded in a 96-well plate at a density of 3,000 cells/well. Five repeats were set. 24, 48, and 72 h after transfection, 10 μLMTT (sigma, United States) was added to each well and then incubated at 37°C for 4 h. Discarded the supernatant and added dimethyl sulfoxide (DMSO, 150 μL per well) to dissolve the purple crystals. Then, the absorbance of each well was measured with a microplate reader (FLUOstar Omega, BMG, Germany) at 492 nm and the cell proliferation curves were plotted.

### Colony Formation Assay

24 h after transfection, MKN-45 and SGC-7901 cells were seeded in a 12-well plate at a density of 2000 cells/well and cultured for 7–10 days. Cell colonies were fixed with 4% paraformaldehyde for 15 min and stained with 0.1% crystal violet for 30 min. After washing out the excess dye twice with phosphate-buffered saline (PBS), photographed the stained cell clones and recorded the number of colonies (>10 cells) to analyze the cell cloning efficiency with Quantity One® software (Bio-Rad, United States).

### Cell Cycle Analysis

24 h after transfection, MKN-45 and SGC-7901 cells were harvested by trypsinization, the cells were washed twice with PBS, and fixed with 70% ice-cold ethanol at 4°C overnight. After two more washes, the cells were incubated with 0.1 mg/ml RNase A and 0.05 mg/ml propidium iodide (Sigma, United States) for 15 min at room temperature. The distribution of the cell cycle stages was examined by flow cytometer (BD, United States).

### Cell Apoptosis Analysis

48 h after transfection, MKN-45 and SGC-7901 cells were harvested by trypsinization, the cells were washed twice with PBS and treated with Annexin V-FITC/PI Apoptosis Detection Kit (7 sea, Shanghai, China) according to the manufacturer’s instructions. Flow cytometry was used to detect stained cells and analyze the level of apoptosis.

### Tumorigenesis Experiment in Nude Mice

MKN-45 cells were infected with a lentiviral vector with luciferase, and also infected with lentiviral vector that knocks down POLR2A (Lv-shPOLR2A, GenePharma, Shanghai, China) and its control (Lv-Control, GenePharma, Shanghai, China) according to the manufacturer’s instructions, then a stable cell line knocked down POLR2A and its control were constructed. Four-week-old male nude mice were purchased from the Experimental Animal Center of Xi’an Jiaotong University and were injected subcutaneously at 1 × 10^7^ cells/mouse. In order to eliminate individual differences, we injected cells of Lv-Control and Lv-shPOLR2A subcutaneously on both sides of the mice’s groin. Tumor growth was observed every 5 days. Four weeks after the injection, the mice were intraperitoneally injected with D-luciferin potassium to stimulate the expression of luciferase (MedChemExpress, United States), anesthetized with isoflurane, and photographed by bioluminescence imaging system (Xenogen, United States) to observe the growth of tumors in living mice by observing the expression of luciferase. The mice were euthanized by cervical dislocation method, the tumors were taken out to measure the volume and weight, and then divided into two parts for RNA and protein extraction. This study was approved by the Biomedical Ethics Committee of the Medical Department of Xi'an Jiaotong University.

### Transwell Migration Assay

Migration assays were performed using transwell chambers with a porous polycarbonate filter (8.0 mm; Merck Millipore, Germany) and inserted into a 24-well plate. Cells in 200 μl of serum-free medium (3 × 10^4^ cells) were added to the upper chamber, and 600 μl of 10% FBS medium was added to the lower chamber. The 24-well plate was incubated in a 5% CO2 cell incubator at 37°C for 24 h. At room temperature, the chambers were fixed with 4% paraformaldehyde for 15 min, and 0.1% crystal violet stained for 30 min. Cells which did not migrate through the wells were wiped by a cotton swab. A microscope imaging system (Nikon, Japan) was used to take pictures to determine the migrating cells.

### Chromatin Immunoprecipitation and ChIP-qRT-PCR

ChIP was conducted as described (Kyng et al., 2003). In brief, MKN-45 and SGC-7901 cells were cross-linked with 1% formaldehyde for 15 min at room temperature and quenched with glycine (125 mmol/L). The nuclear lysate were sonicate by a cell lyser so that the chromatin was sonicated into a fragment of approximately 200 bp. The lysate was divided into two parts and incubated with 5 μg of anti-POLR2A or IgG antibody (Abcam, United Kingdom) at 4°C overnight. DNA-protein complexes were captured by Dynabeads Protein G (Invitrogen, United States) and eluted in TE buffer at 65°C. Decrosslinking was performed at 65°C for 8 h. DNA was extracted using the QIA Rapid PCR Purification Kit (Qiagen, Germany) according to the manufacturer’s instructions, and the DNA was analyzed by qRT-PCR with gene-specific primers. The primer sequences used for ChIP-qRT-PCR are listed in Supplementary Table S6.

### Statistical Analysis

Unless otherwise stated, all experiments were performed at least in triplicate. All statistical analyses were performed using SPSS Statistics 18.0 (Chicago, IL, United States). Datas were expressed as the mean ±SEM of at least three independent experiments, and Student t test was used to calculate the statistical significance of the differences between the groups. All tests were double-sided, and *p* < 0.05 was considered statistically significant.

## Results

### POLR2A Was Highly Expressed in GC Tissues and Showed Differential Expression in GC Cell Lines

**FIGURE 1:**
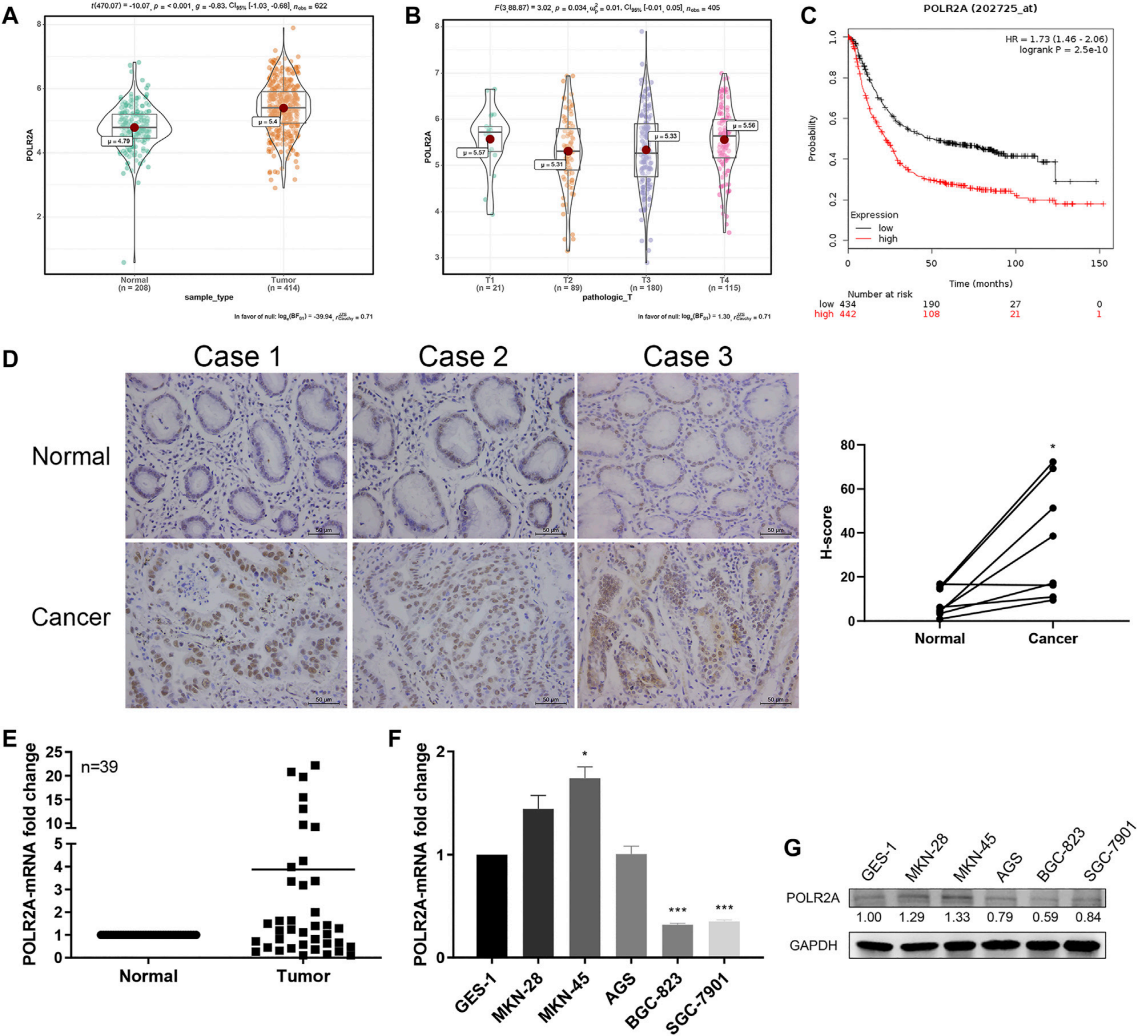
POLR2A was highly expressed in GC tissues and showed differential expression in GC cell lines. **(A)** Bioinformatics analysis of POLR2A expression in GC (*n* = 414) and normal tissues (*n* = 208) based on TCGA data. **(B)** The expression of POLR2A analyzed by TCGA data in T-stage GC tissue samples. **(C)** Kaplan-Meier Plotter based on the expression of POLR2A in GC tissue from the TCGA database. **(D)** Representative images of IHC of POLR2A in paired GC and adjacent normal tissue samples. **(E)** POLR2A mRNA expression in 39 pairs of GC and adjacent normal tissues. **(F,G)** mRNA and protein expression of POLR2A in GC cell lines (MKN-28, MKN-45, AGS, BGC-823 and SGC-7901) and gastric mucosal epithelial cells (GES-1). *p < 0.05; **p < 0.01; ***p < 0.001.

The Cancer Genome Atlas (TCGA) data showed that the expression of POLR2A in GC samples was significantly higher than that of normal samples (Figure 1A). Its expression was significantly related to the size or depth of invasion of the primary GC (T stage, Figure 1B). Patients with high expression of POLR2A had a lower survival rate (Figure 1C). We examined the expression of POLR2A at mRNA and protein levels in 39 GC patients’ GC tissue samples and adjacent normal (non-tumor) tissue samples by qRT-PCR and IHC staining. The results showed that the expression of POLR2A in GC tissues was significantly higher than that in adjacent normal tissues (Figures 1D,E). The mRNA and protein levels of POLR2A in established GC cell lines were detected by qRT-PCR and Western Blotting. However, compared to normal gastric epithelial cells GES-1, POLR2A was highly expressed in MKN-28 and MKN-45 cells, not significantly altered in AGS cells, and significantly low expressed in BGC-823 and SGC-7901 cells (Figures 1F,G).

### POLR2A Promoted the Proliferation of GC Cells *In Vitro*

**FIGURE 2:**
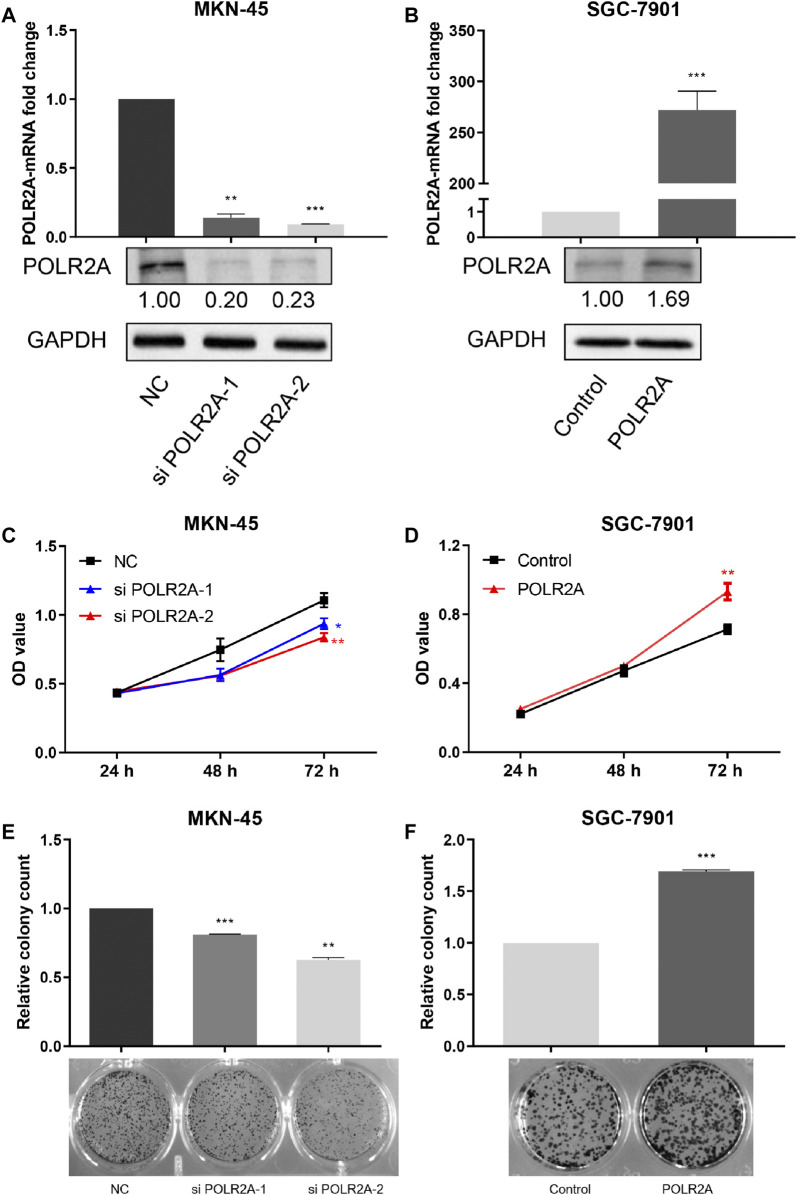
POLR2A promoted the proliferation of GC cells *in vitro*. **(A,B)** POLR2A mRNA and protein expression in MKN-45 cells transfected with POLR2A siRNAs and SGC-7901 cells transfected with POLR2A overexpression plasmid. The mRNA and protein expression of POLR2A in the two cells was detected by qRT-PCR and Western Blotting. **(C,D)** The effect of knockdown/overexpression of POLR2A on the viability of MKN-45/SGC-7901 cells measured by MTT assay. **(E,F)** Colony formation assay 7–10 days after knockdown/overexpression of POLR2A in MKN-45/SGC-7901 cells. *p < 0.05; **p < 0.01; ***p < 0.001.

In order to examine the role of POLR2A in the progression of GC cells, siRNAs targeting POLR2A and its negative control were transfected into MKN-45 cells, and the POLR2A overexpression plasmid and its control empty vector were transfected into SGC-7901 cells. qRT-PCR and Western Blotting were performed to detect the expression of POLR2A at the mRNA and protein levels. Our data showed that, in MKN-45 cells transfected with POLR2A siRNAs, the mRNA and protein levels of POLR2A were significantly down-regulated, while SGC-7901 cells transfected with the POLR2A overexpression plasmid, the mRNA and protein levels of POLR2A were significantly up-regulated, which suggested that siRNAs and overexpression plasmids were successfully constructed (Figures 2A,B). In order to analyze the function of POLR2A in GC cell proliferation, MTT and colony formation assays were performed. MTT analysis showed that knockdown of POLR2A in MKN-45 cells significantly inhibited cell viability at 72 h, while overexpression of POLR2A in SGC-7901 cells significantly improved cell viability at 72 h (Figures 2C,D). Consistent results were also observed in the colony formation assay. As shown in Figures 2E,F, knockdown of POLR2A significantly reduced MKN-45 cell colony formation, and overexpressing POLR2A markedly increased colony formation in SGC-7901 cells. These data indicated that POLR2A promoted the vitality and colony forming ability of GC cells, that is, POLR2A promoted the proliferation of GC cells *in vitro*.

### POLR2A Promoted the Overall Progression of GC Cell Cycle

**FIGURE 3:**
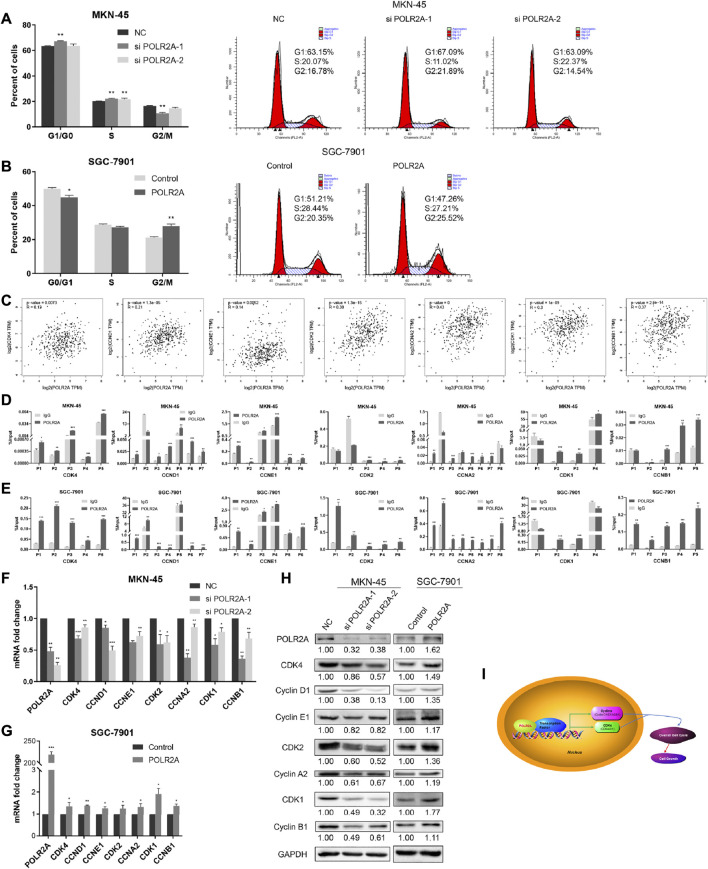
POLR2A promoted the overall progression of GC cell cycle. **(A,B)** Cell cycle changes of MKN-45/SGC-7901 cells that knock down/overexpress POLR2A was measured by flow cytometry. **(C)** The correlation between POLR2A and cyclins/CDKs mRNA expression predicted by the GEPIA database. **(D,E)** Whether the POLR2A antibody captures the target DNA fragment was detected by ChIP-qRT-PCR. **(F,G)** The mRNA level changes of all cyclins and CDKs after knockdown/overexpression of POLR2A in MKN-45/SGC-7901 cells. **(H)** The protein level changes of all cyclins and CDKs after knockdown/overexpression of POLR2A in MKN-45/SGC-7901 cells. **(I)** Schematic diagram of ChIP-qRT-PCR primer design. *p < 0.05; **p < 0.01; ***p < 0.001.

Since cell proliferation was regulated by the cell cycle, we wondered whether POLR2A affects GC cell proliferation by affecting the cycle checkpoint. Flow cytometry was employed to detect the distribution of each phase of the GC cell cycle. Our data showed that knockdown of POLR2A significantly increased the distribution of G1 and S phases in MKN-45 cells, while M phase decreased. Moreover, overexpressing POLR2A decreased the percentage of G1 phase in SGC-7901 cells, and M phase increased (Figures 3A,B). We found that the influence of POLR2A on the GC cell cycle was not reflected in a certain phase, since POLR2A was involved in transcription, we guessed whether POLR2A played a transcriptional regulatory role on all cyclins and cyclin-dependent kinases (CDKs), thereby promoted the overall progression of cell cycle. In order to verify our conjecture, first, the GEPIA database was used to predict the correlation between POLR2A and all cyclins/CDKs at the mRNA expression level. The results showed a significant positive correlation between them (Figure 3C). Then, we searched molecules in the promoter region of cyclins (CCNA2/CCNB1/CCND1/CCNE2) and CDKs (CDK1/CDK2/CDK4) by the UCSC database, and found that POLR2A existed in all (Supplementary Figure S1). Next, primers were designed based on the binding sites of POLR2A and their promoter regions, and ChIP experiments was performed to capture the DNA fragments that bind to the POLR2A antibody and verified by qRT-PCR. Experiment results showed that POLR2A could capture at least one DNA fragment at the binding site of each molecule (Figures 3D,E).

The binding effect of POLR2A with the promoter region of cyclins/CDKs was clarified, so did POLR2A affect their expression? qRT-PCR and Western Blotting were applied to detect the changes of cyclins and CDKs. The results showed that the mRNA and protein expression levels of cyclins and CDKs were down-regulated in MKN-45 cells after knockdown of POLR2A, and up-regulated in SGC-7901 cells after overexpressing POLR2A (Figures 3F–H). Our data demonstrated that POLR2A bound to the promoter regions of cyclins and CDKs to promote their transcription, thereby causing the overall progression of all stages of the cell cycle and promoting cell proliferation (Figure 3I).

### POLR2A Inhibited the Apoptosis of GC Cells

**FIGURE 4:**
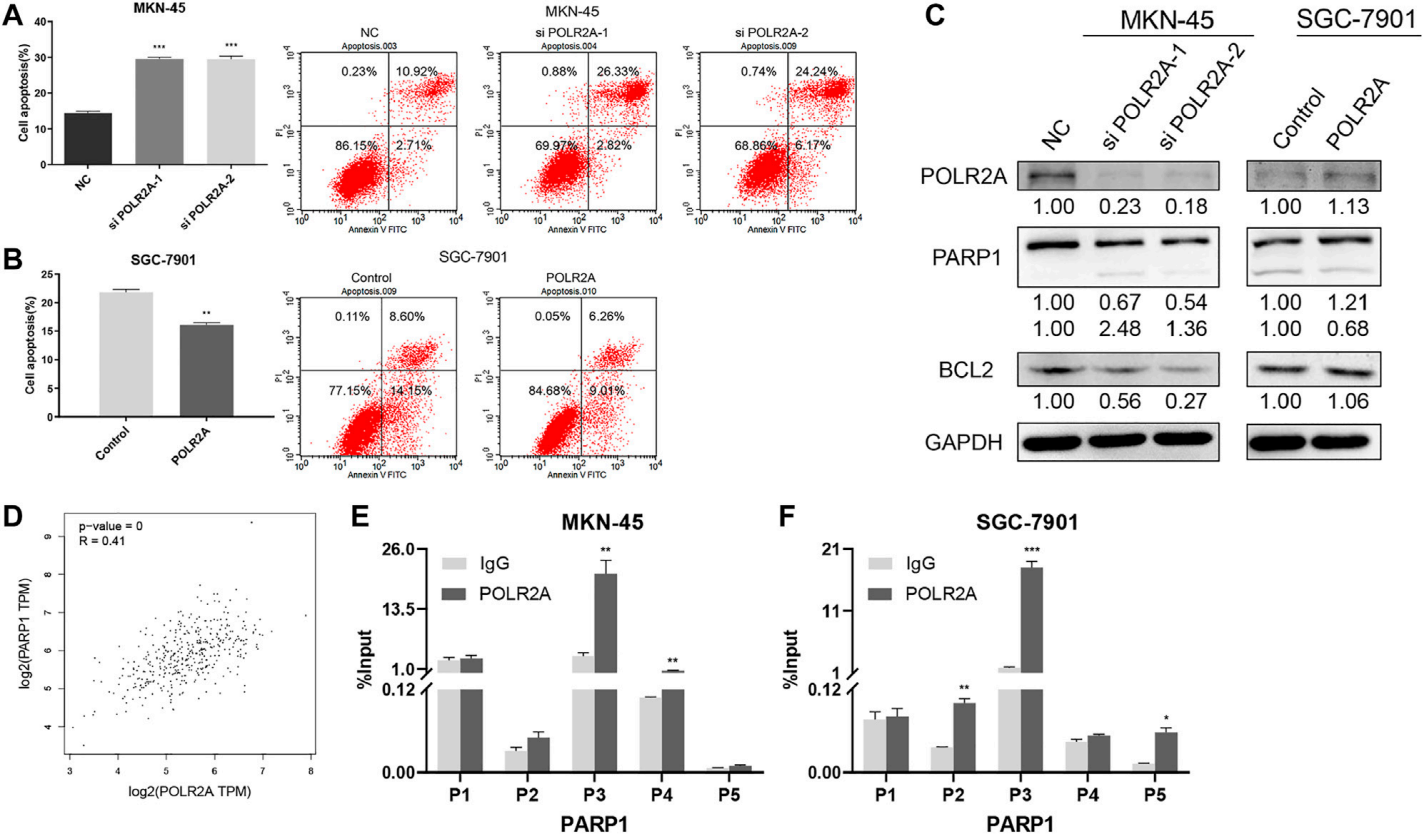
POLR2A inhibited the apoptosis of GC cells. **(A,B)** Cell apoptosis of MKN-45/SGC-7901 cells that knock down/overexpress POLR2A was measured by flow cytometry. **(C)** The protein level changes of apoptosis-related molecules after knockdown/overexpression of POLR2A in MKN-45/SGC-7901 cells. **(D)** The correlation between POLR2A and PARP1 mRNA expression predicted by the GEPIA database. **(E,F)** Whether the POLR2A antibody captures the target DNA fragment was detected by ChIP-qRT-PCR. *p < 0.05; **p < 0.01; ***p < 0.001.

Apoptosis plays a considerable role in the development of cancer. To investigate whether POLR2A affected apoptosis of GC cells, flow cytometry was performed, and Western Blotting was applied to detect changes in apoptosis-related proteins. The results showed that apoptosis was increased in MKN-45 cells after knockdown of POLR2A. In addition, knockdown of POLR2A in MKN-45 cells inhibited the protein expression of anti-apoptotic molecules poly ADP-ribose polymerase 1 (PARP1) and B-cell lymphoma 2 (BCL2). At the same time, apoptosis was reduced in SGC-7901 cells after overexpressing POLR2A, and PARP and BCL2 expression were significantly up-regulated (Figures 4A–C). These results suggested that POLR2A inhibited the apoptosis in GC cells.

PARP1 was an anti-apoptotic molecule, was POLR2A involved in PARP transcriptional regulation? In order to verify this conjecture, the GEPIA database was applied to analyze the correlation between POLR2A and PARP1 mRNA expression, as shown in Figure 4D, there was a significant positive correlation between them. Then we searched PARP promoter region molecules through the UCSC database, and found that POLR2A existed (Supplementary Figure S2). The experimental results of ChIP-qRT-PCR in MKN-45 and SGC-7901 cells also showed that POLR2A captured DNA fragments at the binding site of PARP1 (Figures 4E,F). The above results suggested that POLR2A may inhibit apoptosis through transcriptional regulation of PARP1.

### POLR2A Promoted the Proliferation of GC Cells *In Vivo*

**FIGURE 5:**
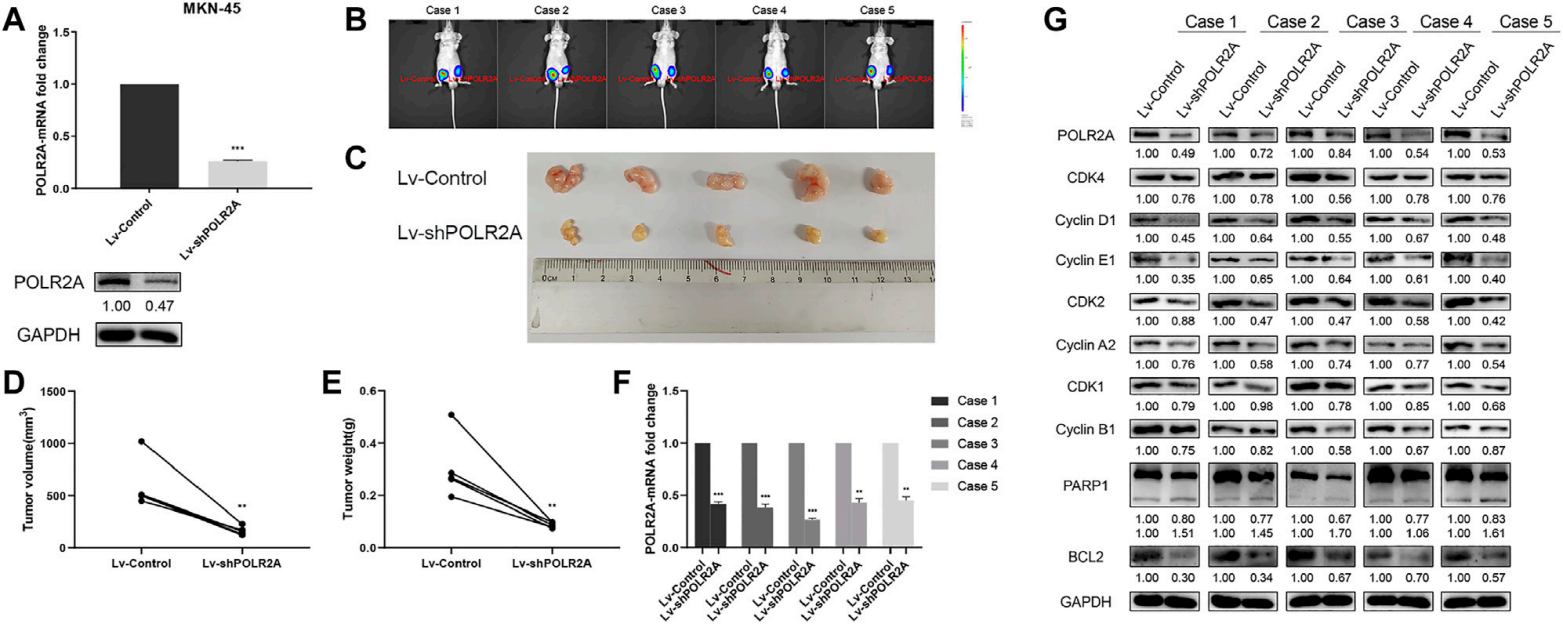
POLR2A promoted the proliferation of GC cells *in vivo*. **(A)** The mRNA and protein expression of POLR2A in MKN-45 cells infected with Lv-shPOLR2A. **(B)** Tumor growth presented by bioluminescence imaging system. The right side of the mice groin was the Lv-Control group, and the left side was the Lv-POLR2A group. **(C)** The tumors were removed to observe its size. **(D,E)** The volume and weight of tumors. **(F)** The mRNA expression of POLR2A in tumors. **(G)** The protein expression of POLR2A and cycle and apoptosis-related molecules in tumors. *p < 0.05; **p < 0.01; ***p < 0.001.

In order to explore the role of POLR2A in the progression of GC *in vivo*, we successfully constructed a stable MKN-45 cell line that knocked down POLR2A and its control (Figure 5A), and then performed tumorigenesis experiment in nude mice. The two groups of cells were injected subcutaneously into the groin on both sides of nude mice to observe tumor growth. Four weeks after the injection, the bioluminescence imaging system showed that the tumor volume of Lv-shPOLR2A was significantly smaller than that of Lv-Control (Figure 5B). The volume and weight of the removed solid tumor were measured, and the results showed that the tumor volume and weight of Lv-shPOLR2A were significantly smaller than that of Lv-Control (Figures 5C–E). The qRT-PCR and Western Blotting results of solid tumors showed that compared with Lv-Control, the expression of POLR2A of Lv-shPOLR2A was significantly down-regulated at the RNA and protein levels (Figures 5F,G). We also tested the expression of the cycle and apoptosis-related molecules involved in the aforementioned *in vitro* experiments at the protein level, and the results were consistent with the previous *in vitro* experiments. As shown in Figure 5G, compared to Lv-Control, the protein expression levels of cyclins and CDKs of Lv-shPOLR2A were down-regulated, and the protein expression levels of PARP and BCL2 were also down-regulated. These findings were consistent with the *in vitro* results, indicated that POLR2A also had the effect of promoting GC proliferation *in vivo*.

### POLR2A Promoted the Migration of GC Cells

**FIGURE 6:**
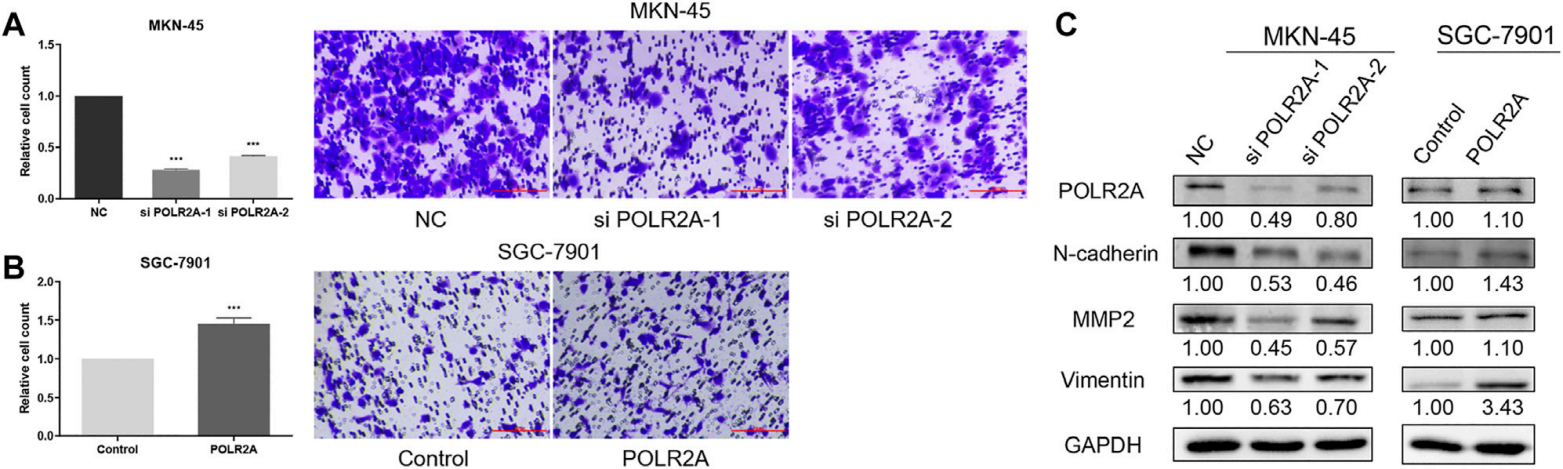
POLR2A promoted the migration of GC cells. **(A,B)** Cell migration of MKN-45/SGC-7901 cells that knock down/overexpress POLR2A was measured by transwell migration assay. **(C)** The protein level changes of migration-related molecules after knockdown/overexpression of POLR2A in MKN-45/SGC-7901 cells. *p < 0.05; **p < 0.01; ***p < 0.001.

To investigate the effect of POLR2A on the migration of GC cells, we employed the transwell migration assay. The migration of MKN-45 cells was inhibited after knockdown of POLR2A, while the migration was promoted after POLR2A overexpressed in SGC-7901 cells (Figures 6A,B). The levels of Matrix Metallopeptidase 2 (MMP2), Vimentin, and N-cadherin were decreased in POLR2A-knockdown MKN-45 cells, while their expression levels were up-regulated in SGC-7901 cells after overexpressing POLR2A (Figure 6C). The above results indicated that POLR2A had enhancement in the migration of GC cells.

## Discussion

More and more evidences show that POLR2A plays an oncogene in tumors. Highly expressed POLR2A was associated with the poor prognosis of TNBC patients, inhibition of POLR2A would reduce tumor growth (Kurokawa et al., 2017). In CRC, silencing POLR2A led to inhibition of cell proliferation, cycle arrest and increased apoptosis (Liu et al., 2015; Gao and Liu, 2019). Similarly, POLR2A was highly expressed in acute myeloid leukemia (AML) cells and tissue samples, and positively correlated with the malignant proliferation of leukemia cells. AML patients with high POLR2A expression also had a poor prognosis (Radhakrishnan and Gartel, 2006). Besides, studies had reported that POLR2A gene polymorphism was associated with lower survival outcomes in patients with non-small cell lung cancer (Sainsbury et al., 2015). In addition, Zhang et al. discovered the key potential transcription axis of CTCF/POLR2A—SYNJ2/INPP5B in metabolic programs based on the ChIP-seq data set, speculated that CTCF/POLR2A could directly dysregulate SYNJ2 levels and that increased SYNJ2 would affect HCC development via metabolic perturbation pathways (Zhang et al., 2021). The above studies consistently show that POLR2A promotes tumor growth and is related to poor prognosis. However, as far as we know, the expression and role of POLR2A in GC have not been reported. We used bioinformatics to predict the survival rate of POLR2A in GC and found that patients with high POLR2A expression have a lower survival rate. We also proved that POLR2A was highly expressed in GC tissues through bioinformatics and experiments, which is consistent with the results of studies in other tumors.

The basic biological processes of cells, such as survival, growth and differentiation, are inseparable from transcription (Saldi et al., 2016). Tumor cells require higher levels of transcription to meet their rapid proliferation characteristics (Radhakrishnan and Gartel, 2006). POLR2A, as the core of the transcription mechanism, is considered to be an essential transcriptional oncogene and anti-apoptotic factor, which could maintain the rapid growth and apoptosis resistance of tumor cells (Schafer, 1998; Serra et al., 2019). Therefore, POLR2A is highly expressed in tumor tissues compared with normal tissues. Our results also confirmed this. Furthermore, the biological function experiments of GC cells *in vitro* showed that the proliferation of MKN-45 cells was inhibited after knockdown POLR2A, the overall cell cycle was suppressed, the expression of cyclins and CDKs was down-regulated, apoptosis increased, and the expression of anti-apoptotic proteins PARP and BCL2 is down-regulated, and the overexpression of POLR2A in SGC-7901 cells has the opposite effect of knockdown POLR2A in MKN-45 cells, these results indicated that POLR2A promoted the proliferation of GC by advancing the cell cycle and inhibiting apoptosis. *In vivo* experiments in mice further confirmed the promoting effect of POLR2A on GC. Apart from this, we also found that POLR2A promoted the migration of GC cells, and up-regulated the expression of N-cadherin, MMP2 and Vimentin, which were involved in epithelial-mesenchymal transition (EMT) (Nfonsam et al., 2019; Xu et al., 2021). However, the specific molecular mechanism needs to be further studied.

The cell cycle of eukaryotic cells is a relatively complex process. The changes in the biochemical and morphological structure of the cell, as well as the transition between adjacent phases, are carried out in an orderly manner under the strict control of the cell itself and environmental factors (Villicana et al., 2014; Song et al., 2017). The cell cycle process is driven by an evolutionary conserved central mechanism. Cyclins and CDKs(Cyclin-CDK) form the core of the cell cycle control system (Wenzel and Singh, 2018; Xu et al., 2019). The periodic formation and degradation of Cyclin-CDK complex triggers the emergence of specific events in the cell cycle process, and promotes the irreversible transformation of key processes from G1 phase to S phase, G2 phase to M phase, and mid to late phases. Therefore, the correct cooperation between them is essential to ensure the orderly progression of the complete cell cycle. Additionally, the detection point monitors important events and malfunctions that occur in the cell cycle (Yoo et al., 2017) Our study demonstrates that POLR2A has an overall effect on the cell cycle. The cell cycle that knockdown POLR2A shows an overall blockage phenomenon, while an overall advancement of POLR2A overexpression, indicating that POLR2A does not only act on a certain checkpoint of the cell cycle, but also promotes each phase of the cell cycle. Furthermore, our chromatin immunoprecipitation experiments confirmed that POLR2A could capture DNA fragments of cyclins (CCNA2/CCNB1/CCND1/CCNE1) and CDKs (CDK1/CDK2/CDK4) genes in each phase. As we all know, transcription is not an independent process. When RNAP Ⅱ initiates transcription, it needs the participation of universal transcription factors to form an active transcription complex (Zhao et al., 2017; Yu et al., 2019). POLR2A is the largest subunit of RNAP Ⅱ, its role in transcription regulation is by recruiting transcription factors to the promoter region of target genes to promote the transcription of target genes, instead of directly playing the role of the transcription factor (Zhou et al., 2016).

In summary, we report here the high expression of POLR2A in GC and its promotion to GC *in vitro* and *in vivo*. POLR2A promoted the overall process of the GC cell cycle by regulating the transcription of cyclins and CDKs. In addition, POLR2A inhibited GC cell apoptosis and promoted GC cell migration. Our data suggest that POLR2A plays an oncogene role in the progression of GC and is expected to become a potential therapeutic target for GC. This study explains the role of POLR2A in the progession of GC, and its specific molecular mechanism and targeted drugs in GC need to be further studied.

## Data Availability

The original contributions presented in the study are included in the article/Supplementary Material, further inquiries can be directed to the corresponding authors.

## Supplementary Material

Supplementary Figure S1 Transcriptional regulatory molecules in the promoter region of cyclins and CDKs searched by the UCSC database.

Supplementary Figure S2 Transcriptional regulatory molecules in the promoter region of PARP1 searched by the UCSC database.
