# Supplementary material for: POLR2A Promotes the Proliferation of Gastric Cancer Cells by Advancing the Overall Cell Cycle Progression
Source: Front Genet. 2021 Nov 25;12:688575. doi: 10.3389/fgene.2021.688575 (PMC8655910; doi:10.3389/fgene.2021.688575)
Supplement: Supplementary file 1 [file DataSheet1.docx]

**Table S1. Correlation of POLR2A expression with clinicopathologic characteristics in GC patients**

| **Characteristics** | **No.of patients** | **POLR2A expression** | | **P value** |
| --- | --- | --- | --- | --- |
|  |  | **Low** | **High** |  |
| Gender |  |  |  | 0.223 |
| Male | 30 | 7 | 23 |  |
| Female | 9 | 4 | 5 |  |
|  |  |  |  |  |
| Age |  |  |  | 0.298 |
| ≥60 | 21 | 7 | 14 |  |
| ＜60 | 18 | 9 | 9 |  |
|  |  |  |  |  |
| Histologic grade |  |  |  | 0.94 |
| Well differetiated | 0 | 0 | 0 |  |
| Moderately differetiated | 10 | 4 | 6 |  |
| Poorly differetiated | 29 | 12 | 17 |  |
|  |  |  |  |  |
| TNM stage |  |  |  | 0.556 |
| Ⅰ | 0 | 0 | 0 |  |
| Ⅱ | 11 | 5 | 6 |  |
| Ⅲ | 23 | 8 | 15 |  |
| Ⅳ | 5 | 3 | 2 |  |
|  |  |  |  |  |
| LN metastasis |  |  |  | 0.082 |
| Absence | 4 | 0 | 4 |  |
| Presence | 35 | 16 | 19 |  |
|  |  |  |  |  |
| Patient Number | 39 | 16 | 23 |  |

Association between POLR2A expression and clinicopathological characteristics was analyzed by Kruskal-Wallis test.

**Table S2. All antibodies**

| **Antibody** | **Brand** | **Catalog number** | **Host species** | **Clone** | **Dilution** | **Duration of the incubation** |
| --- | --- | --- | --- | --- | --- | --- |
| POLR2A | Abcam | ab817 | Mouse | 8WG16 | IHC : 1:100 | 4 ℃，overnight |
| GAPDH | Proteintech | 60004-1-Ig | Mouse | 1E6D9 | WB : 1:20000 | 4 ℃，overnight |
| POLR2A | Proteintech | 20655-1-AP | Rabbit | Polyclonal | WB : 1:500 | 4 ℃，overnight |
| CDK4 | Proteintech | 11026-1-AP | Rabbit | Polyclonal | WB : 1:1000 | 4 ℃，overnight |
| Cyclin D1 | Proteintech | 60186-1-Ig | Mouse | 2G3G5 | WB : 1:5000 | 4 ℃，overnight |
| Cyclin E1 | Proteintech | 11554-1-AP | Rabbit | Polyclonal | WB : 1:500 | 4 ℃，overnight |
| CDK2 | Proteintech | 10122-1-AP | Rabbit | Polyclonal | WB : 1:10000 | 4 ℃，overnight |
| Cyclin A2 | Proteintech | 18202-1-AP | Rabbit | Polyclonal | WB : 1:2000 | 4 ℃，overnight |
| CDK1 | Proteintech | 19532-1-AP | Rabbit | Polyclonal | WB : 1:1000 | 4 ℃，overnight |
| Cyclin B1 | Proteintech | 55004-1-AP | Rabbit | Polyclonal | WB : 1:1000 | 4 ℃，overnight |
| PARP | Cell Signaling Technology | 9532S | Rabbit | 46D11 | WB : 1:1000 | 4 ℃，overnight |
| BCL2 | Proteintech | 12789-1-AP | Rabbit | Polyclonal | WB : 1:1000 | 4 ℃，overnight |
| N-cadherin | Proteintech | 22018-1-AP | Rabbit | Polyclonal | WB : 1:2000 | 4 ℃，overnight |
| MMP2 | Proteintech | 10373-2-AP | Rabbit | Polyclonal | WB : 1:500 | 4 ℃，overnight |
| Vimentin | Proteintech | 10366-1-AP | Rabbit | Polyclonal | WB : 1:2000 | 4 ℃，overnight |

**Table S3. Sequence-specific primers for qRT-PCR**

| **Primer name** | **Sequence** |
| --- | --- |
| GAPDH F | GGAGCGAGATCCCTCCAAAAT |
| GAPDH R | GGCTGTTGTCATACTTCTCATGG |
| POLR2A F | GCGGAATGGAAGCACGTTAAT |
| POLR2A R | CCCAGCACAAAACACTCCTC |
| CDK4 F | ATGGCTACCTCTCGATATGAGC |
| CDK4 R | CATTGGGGACTCTCACACTCT |
| CCND1 F | CAATGACCCCGCACGATTTC |
| CCND1 R | CATGGAGGGCGGATTGGAA |
| CCNE1 F | AAGGAGCGGGACACCATGA |
| CCNE1 R | ACGGTCACGTTTGCCTTCC |
| CDK2 F | CCAGGAGTTACTTCTATGCCTGA |
| CDK2 R | TTCATCCAGGGGAGGTACAAC |
| CCNA2 F | GGATGGTAGTTTTGAGTCACCAC |
| CCNA2 R | CACGAGGATAGCTCTCATACTGT |
| CDK1 F | GGATGTGCTTATGCAGGATTCC |
| CDK1 R | CATGTACTGACCAGGAGGGATAG |
| CCNB1 F | TTGGGGACATTGGTAACAAAGTC |
| CCNB1 R | ATAGGCTCAGGCGAAAGTTTTT |

**Table S4. siRNAs sequence**

| **Gene Name** | **Sequence** |
| --- | --- |
| Negative siRNA (NC) sense | UUCUCCGAACGUGUCACGUTT |
| Negative siRNA (NC) antisense | ACGUGACACGUUCGGAGAATT |
| POLR2A siRNA-1 sense | GGAUUCCAUUUGGCUUCAATT |
| POLR2A siRNA-1 antisense | UUGAAGCCAAAUGGAAUCCTT |
| POLR2A siRNA-2 sense | CCAAGCUACUCUCCAACAUTT |
| POLR2A siRNA-2 antisense | AUGUUGGAGAGUAGCUUGGTT |

**Table S5-1. Transfection system (DNA)**

| **Culture Vessel** | **Volume of jetPRIME® Buffer (µl)** | **Amount of DNA (µg)** | **Volume of jetPRIME® reagent (µl)** | **Volume of growth medium (ml)** | **Number of cells at inoculation** |
| --- | --- | --- | --- | --- | --- |
| 96-well | 10 | 0.05 | 0.2 | 0.1 | 3×10^3^ |
| 24-well | 40 | 0.4 | 0.8 | 0.4 | 5×10^4^ |
| 12-well | 80 | 0.8 | 1.6 | 0.8 | 1×10^5^ |
| 6-well | 200 | 2 | 4 | 2 | 2×10^5^ |

**Table S5-2. Transfection system (siRNA)**

| **Culture Vessel** | **Volume of jetPRIME® Buffer (µl)** | **Amount of siRNA (nmol)** | **Volume of jetPRIME® reagent (µl)** | **Volume of growth medium (ml)** | **Number of cells at inoculation** |
| --- | --- | --- | --- | --- | --- |
| 96-well | 10 | 5 | 0.2 | 0.1 | 3×10^3^ |
| 24-well | 40 | 20 | 0.8 | 0.4 | 5×10^4^ |
| 12-well | 80 | 40 | 1.6 | 0.8 | 1×10^5^ |
| 6-well | 200 | 100 | 4 | 2 | 2×10^5^ |

**Table S6. Sequence-specific primers for ChIP-qRT-PCR**

| **Primer name** | **Sequence** |
| --- | --- |
| POLR2A-CDK4-1 F | CCACTGGCTCATATCGAGAG |
| POLR2A-CDK4-1 R | TCTCTGGGGCCGGCCCCAAG |
| POLR2A-CDK4-2 F | GCCCCAGAGATAACACAATG |
| POLR2A-CDK4-2 R | TCAAGGGCGGGAAGTGGGGC |
| POLR2A-CDK4-3 F | TCCGGAGCAGCTGGACGCAG |
| POLR2A-CDK4-3 R | TCCCAGTCGAAGCACCTCCT |
| POLR2A-CDK4-4 F | TCGACTGGGAGGAGGGCGAA |
| POLR2A-CDK4-4 R | CGCCTCGGGCTCCACCCTCT |
| POLR2A-CDK4-5 F | CATACTCTCATTTGTGTCAT |
| POLR2A-CDK4-5 R | TTTGCCCTCAAATGGGCATT |
| POLR2A-CCND1-1 F | TCAGAGGTGTGTTTCTCCCG |
| POLR2A-CCND1-1 R | AAAAAATAAAATAAAATAAA |
| POLR2A-CCND1-2 F | TTTATTTTTTGAGCGAGCGC |
| POLR2A-CCND1-2 R | GCGACTGCATCTTCTTTCAT |
| POLR2A-CCND1-3 F | ATGCAGTCGCTGAGATTCTT |
| POLR2A-CCND1-3 R | GGGGTGAGGTGGAGGTGGCT |
| POLR2A-CCND1-4 F | ACCTCACCCCCTAAATCCCG |
| POLR2A-CCND1-4 R | AAACGCCGGGAGCAGCGAGG |
| POLR2A-CCND1-5 F | CCCGGCGTTTGGCGCCCGCG |
| POLR2A-CCND1-5 R | AGCGTGCGGACTCTGCTGCT |
| POLR2A-CCND1-6 F | TCCGCACGCTCCGGCGAGGG |
| POLR2A-CCND1-6 R | CCGGTCGTTGAGGAGGTTGG |
| POLR2A-CCND1-7 F | CAACGACCGGGTGCTGCGGG |
| POLR2A-CCND1-7 R | GAAGGGAGGGGGGTGAGTAG |
| POLR2A-CCNE1-1 F | GTGTGGATTTGACCCTTATG |
| POLR2A-CCNE1-1 R | TCTGACTCTGGGATTCCCTT |
| POLR2A-CCNE1-2 F | CAGAGTCAGAAAGGTCTTCA |
| POLR2A-CCNE1-2 R | GCGCCTGTGCCTTGGCCTAG |
| POLR2A-CCNE1-3 F | GCACAGGCGCGGTGACCTTG |
| POLR2A-CCNE1-3 R | CGTGGCTGGCGCGGGTGGAA |
| POLR2A-CCNE1-4 F | GCCAGCCACGCGGCTTTTTG |
| POLR2A-CCNE1-4 R | CGCAGGGACGGGGAATCAGG |
| POLR2A-CCNE1-5 F | CGTCCCTGCGCCTCGCGGGC |
| POLR2A-CCNE1-5 R | CACCGCGCTGGCGGCCGCGC |
| POLR2A-CCNE1-6 F | CAGCGCGGTGTAGGGGGCAG |
| POLR2A-CCNE1-6 R | CCCGCGACCCGGCCGCACCC |
| POLR2A-CDK2-1 F | GGAGGAGAGGAAAAAGGAAA |
| POLR2A-CDK2-1 R | CTGGTTTAATTCACTCTCCC |
| POLR2A-CDK2-2 F | ATTAAACCAGGAACTTCTCA |
| POLR2A-CDK2-2 R | CAATCCTGGGGAAAATATGT |
| POLR2A-CDK2-3 F | GGCTCTGACGTTGACCAATA |
| POLR2A-CDK2-3 R | CGGGGGCCGAGGAGGGGGAA |
| POLR2A-CDK2-4 F | TCGGCCCCCGAGAGCCAGGG |
| POLR2A-CDK2-4 R | CCACTCACGTGTCCAGGCGG |
| POLR2A-CDK2-5 F | ACGTGAGTGGCCTCTGTACC |
| POLR2A-CDK2-5 R | ATTCCACACCACCCTATACT |
| POLR2A-CCNA2-1 F | ATTAAACTTTCTGGGACAAA |
| POLR2A-CCNA2-1 R | TGCTCCACCCTCGGGCGACA |
| POLR2A-CCNA2-2 F | GGGTGGAGCAACAGAGCAAA |
| POLR2A-CCNA2-2 R | CATCTGCCTGTACATAATGG |
| POLR2A-CCNA2-3 F | CAGGCAGATGCCCCCTATAG |
| POLR2A-CCNA2-3 R | ATATCTGCAGGAGGGTGGGT |
| POLR2A-CCNA2-4 F | CTGCAGATATCCCGCATCCC |
| POLR2A-CCNA2-4 R | CTGCGCCGGGGCCTGCGACC |
| POLR2A-CCNA2-5 F | CCCGGCGCAGAGTTGCCCAA |
| POLR2A-CCNA2-5 R | GCCTGCCTGCGCCTTTCGCA |
| POLR2A-CCNA2-6 F | GCAGGCAGGCACTGCCCAGC |
| POLR2A-CCNA2-6 R | GTTTGTTTCTCCCTCCTGCC |
| POLR2A-CCNA2-7 F | AGAAACAAACTGGCTGGGGC |
| POLR2A-CCNA2-7 R | AGTCACTTAAGCTAACTAGA |
| POLR2A-CCNA2-8 F | TTAAGTGACTTTTTCCAAAG |
| POLR2A-CCNA2-8 R | TGTTAAGATGACATTACAGT |
| POLR2A-CDK1-1 F | GGAGCGAACAGTAGCTTCCT |
| POLR2A-CDK1-1 R | GACTTTCAAAGCAGCCAATC |
| POLR2A-CDK1-2 F | TTTGAAAGTCTACGGGCTAC |
| POLR2A-CDK1-2 R | CTCGGCCGTCCCCTAGACAC |
| POLR2A-CDK1-3 F | GACGGCCGAGGGCCTCGGAG |
| POLR2A-CDK1-3 R | CACGCCGCCTAACCCCGCCA |
| POLR2A-CDK1-4 F | AGGCGGCGTGGGGGTGGGGG |
| POLR2A-CDK1-4 R | TGCGGTCCCACTTTCCCACC |
| POLR2A-CCNB1-1 F | AAACTGGACAATCTTGAAGT |
| POLR2A-CCNB1-1 R | CATTCTTTCATTTGATCGTT |
| POLR2A-CCNB1-2 F | TGAAAGAATGTCTATTAAAG |
| POLR2A-CCNB1-2 R | CCAGTGCGCCGCCCACCTGG |
| POLR2A-CCNB1-3 F | GGCGCACTGGCTTCACTGCT |
| POLR2A-CCNB1-3 R | ATTGGCCTGTTCGTGGCACT |
| POLR2A-CCNB1-4 F | ACAGGCCAATAAGGAGGGAG |
| POLR2A-CCNB1-4 R | TCGCAGTCCGAAGCGGCTCA |
| POLR2A-CCNB1-5 F | CGGACTGCGAACTAACGCGG |
| POLR2A-CCNB1-5 R | AATATCCGTCCAGTTCTCTT |
| POLR2A-PARP1-1 F | CGGGCCGCTCGGGAGGAGGG |
| POLR2A-PARP1-1 R | AGCATCCCCAAGGACTCGCT |
| POLR2A-PARP1-2 F | TGGGGATGCTCTCGCTGCAT |
| POLR2A-PARP1-2 R | GCGGCTCTGGCCGCTCAGGC |
| POLR2A-PARP1-3 F | CCAGAGCCGCCACCGAACAC |
| POLR2A-PARP1-3 R | AGCCCCGCCTCGGCCCCCAG |
| POLR2A-PARP1-4 F | AGGCGGGGCTTGGGCTGAGC |
| POLR2A-PARP1-4 R | ATCGCTGGGCTCAGGAGTTC |
| POLR2A-PARP1-5 F | GCCCAGCGATCCTCCCACCT |
| POLR2A-PARP1-5 R | TTTGGGGCCGGCGTGGTAGC |
